# Origin of band gaps in graphene on hexagonal boron nitride

**DOI:** 10.1038/ncomms7308

**Published:** 2015-02-19

**Authors:** Jeil Jung, Ashley M. DaSilva, Allan H. MacDonald, Shaffique Adam

**Affiliations:** 1 Graphene Research Centre, Department of Physics, National University of Singapore, 2 Science Drive 3, Singapore 117551, Singapore; 2 Department of Physics, The University of Texas at Austin, 2515 Speedway, C1600 Austin, Austin, Texas 78712-1192, USA; 3 Yale-NUS College, 6 College Avenue East, Singapore 138614, Singapore

## Abstract

Recent progress in preparing well-controlled two-dimensional van der Waals heterojunctions has opened up a new frontier in materials physics. Here we address the intriguing energy gaps that are sometimes observed when a graphene sheet is placed on a hexagonal boron nitride substrate, demonstrating that they are produced by an interesting interplay between structural and electronic properties, including electronic many-body exchange interactions. Our theory is able to explain the observed gap behaviour by accounting first for the structural relaxation of graphene’s carbon atoms when placed on a boron nitride substrate, and then for the influence of the substrate on low-energy *π*-electrons located at relaxed carbon atom sites. The methods we employ can be applied to many other van der Waals heterojunctions.

## Abstract (web summary)

Graphene doesn’t usually have a bandgap but one can appear when the two-dimensional material is placed on a hexagonal boron nitride substrate. Jung *et al*. now develop a theory that indicates that this occurs because the graphene’s carbon atoms structurally relax when placed on boron nitride.

## Graphical abstract

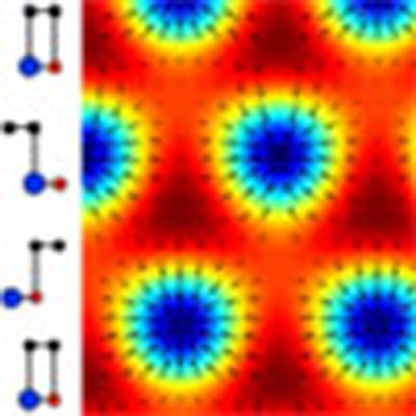

Recent progress in preparing vertical heterojunctions of graphene (G) and hexagonal boron nitride (BN) using either transfer^1^ or growth techniques^2^ has opened a new frontier for exploring both fundamental physics^3^,^4^,^5^ and new device geometries^6^. Experiments have made it clear that G on BN is very flat and that its low-energy electronic states are often very weakly perturbed by the substrate^1^. However, when the honeycomb lattices of G and BN are close to orientational alignment, the electronic coupling strengthens and is readily observed^7^. The source of this variability in behaviour is clearly related to variability in structure. For example, although *ab initio* theory^8^,^9^ predicts substantial gaps ~50 meV when the two honeycomb lattices are identical, any incommensurability due to misorientation or lattice constant mismatch drastically reduces electronic coupling, giving vanishingly small gaps^10^.

Here, we show that the large gaps observed^3^ at the Fermi level of neutral G sheets that are nearly rotationally aligned with a BN substrate are not due solely in terms of the relative orientation-dependent moiré pattern, but require in addition both orientation-dependent structural relaxation of the carbon atoms, as suggested by recent experiments^11^, and nonlocal many-body exchange interactions between electrons. Our theory involves two elements: (i) structural relaxation due to interactions between G and the BN substrate and (ii) an effective Hamiltonian for G’s *π*-electrons, which includes a substrate interaction term that is dependent on the local coordination between G and BN honeycombs. Our main results are that atomic relaxation leads to substantially enhanced gap. The band gap for rotationally aligned layers is only ~1 meV when the honeycomb lattices are held rigid, but increases to ~7 meV when relaxation is allowed. These gaps are further enhanced to ~20 meV, in reasonable agreement with the experiment, when we also account for electron–electron interactions. Moreover, unlike other proposed mechanisms for band gaps in G^12^, ours does not degrade the mobility of G.

## Results

### Moiré patterns and strains

**Figure 1:**
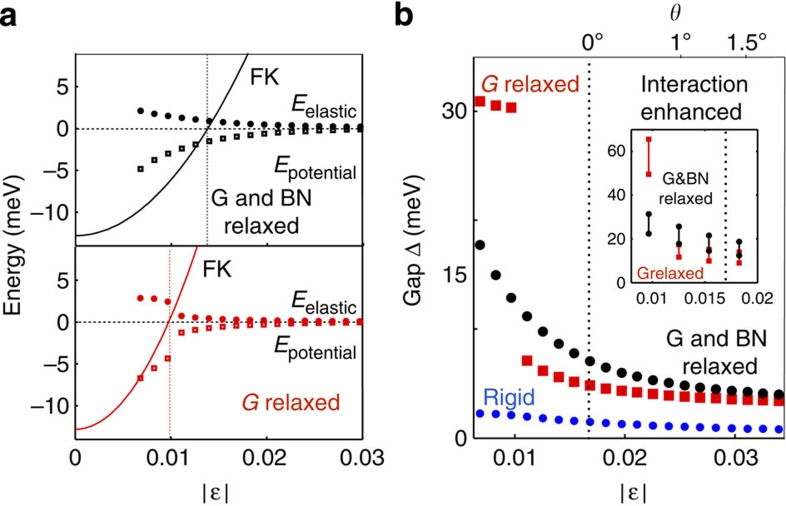
Relaxation strains and band gaps of G on BN. (**a**) Relaxation strain elastic and potential energies for orientation aligned G on BN as a function of *ε* the relative lattice constant difference. The black lines illustrate the case in which only carbon atom positions are allowed to relax (black), whereas the red curve is for the case in which both G and BN layer atoms are allowed to relax. The parabolic curve labelled FK plots the energy difference between an undistorted G sheet and one with a lattice that has expanded to be commensurate with that of the substrate that is discussed in the text. *E*_elastic_ and *E*_potential_ are, respectively, the elastic energy cost and the potential energy gained by straining both G and BN (black) layers, and the G (red) only while keeping the moiré lattice constant fixed. We use *ε*=−0.017 for G on BN in the absence of G lattice expansion. (**b**) Energy gaps including strain effects versus *ε* when G and BN layers are allowed to relax (black) and when only G atoms are allowed to relax (red), when the layers are held rigid at 3.4 Å separation (blue), and when electron–electron interactions are also included (inset). The interaction-enhanced gaps are bracketed by Hartree–Fock calculations that use dielectric constants of 2.5 and 4 to account for screening effects. The *θ* label indicates the one-to-one relation with *l*_M_ when we fix |*ε*|=0.017 and provides an approximate representation of the twist angle dependence.

The summary of our main results is presented in Fig. 1 that shows the lattice relaxation-dependent gap. The *π*-electron Hamiltonian of G/BN can be expressed as the sum of the continuum model Dirac Hamiltonian of an isolated flat G sheet, in which the honeycomb sublattice degree-of-freedom appears as a pseudo spin, and a correction from the interaction with the BN substrate^7^,^9^,^10^,^13^. We employ an approach in which the correction is given by a sublattice-dependent but spatially local operator *H*_M_(**d**) derived from the *ab initio* theory^9^ that depends on the local alignment between G and BN honeycomb lattices *d*. This pseudospin dependent operator that gives rise to the moiré superlattice Hamiltonian is accurately parameterized in ref. 9. (An alternate parameterization that allows spatial variation in the interlayer separation is discussed in the Methods section.)

When both G and BN form rigid honeycomb lattices


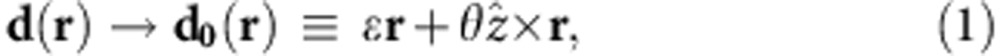


where *ε* is the difference between their lattice constants, *θ* is the difference in their orientations and 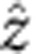 is the direction normal to the G sheet. The two layers establish a moiré pattern in which equivalent alignments repeat periodically on a length scale that, when *ε* and *θ* are small, is long compared with the honeycomb lattice constant. (The moiré lattice vectors **L**_M_ solve **d**(**r**+**L**_M_)=**d**(**r**)+**L**, where **L** is a honeycomb lattice vector.) Since *H*_M_(**d**)=*H*_M_(**d**+**L**), the substrate interaction Hamiltonian has the periodicity of the moiré pattern.

When the honeycomb lattices of the G and BN layers are allowed to relax, **d**(**r**) is no longer a simple linear function of position. We write


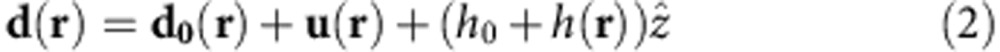


where *h*_0_ is the mean separation between G and BN planes, and the in-plane and vertical strains, **u**(**r**) and *h*(**r**), also have the moiré pattern periodicity. If G/BN systems achieved thermal equilibrium *ε*, *θ*, **u**(**r**) and *h*(**r**) would be determined by minimizing free energy with respect to the positions of atoms in the G layer and in the BN layers close to the surface of the substrate. Evidently, this is not the case since the observed value of *θ* varies in an irreproducible manner. In the following we take the view that because the thermodynamic bias favouring a particular value of *θ* is weak, its observed value is fixed by transfer kinetics. Similarly, the value of *ε*, which can be adjusted only by atomic rearrangements on long length scales, is also likely determined by kinetics and not by equilibrium considerations. On the other hand, given values for *ε* and *θ* minimizing energy with respect to local strains **u**(**r**) and *h*(**r**) require only local atomic arrangements. We therefore view *ε* and *θ* as experimentally measurable system parameters. In practice, *ε* is close to the undisturbed relative lattice constant difference, whereas *θ* varies widely. The ratio of the honeycomb lattice constant to the moiré pattern lattice constant *l*_M_ is *a*/*l*_M_=(*ε*^2^+*θ*^2^)^1/2^. For given values of *θ* and *ε*, *H*_M_(**d**(**r**)) is dependent on strains because of their contribution to equation (2). The strains must therefore be calculated first in order to fix the *θ*, *ε*-dependent *π*-band Hamiltonian of G/BN. As a side remark, we note that **d**(**r**)=(*a*/*l*_M_)**r** is a convenient approximation for the coordination vector that can account for the twist angle dependence through the magnitude of *l*_M_ but ignores the variations in the shape of the moire pattern.

**Figure 2:**
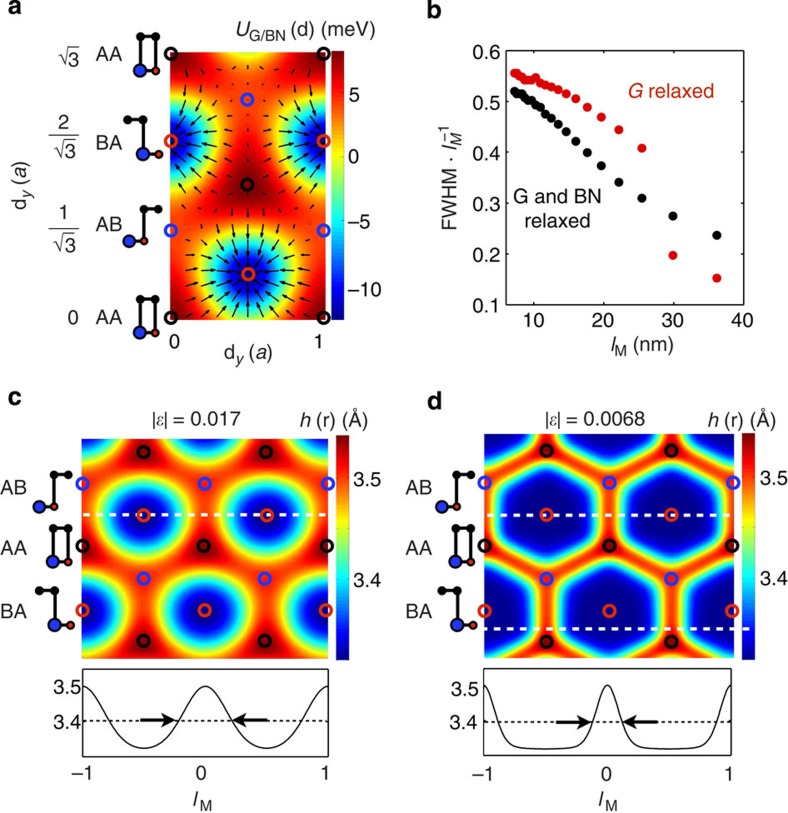
Relaxation strain and degree of commensuration as a function of the moire pattern lattice constant. (**a**) Substrate interaction energy *U*(d) per unit cell area as a function of stacking coordination *d*. The arrows indicate the magnitudes and directions of substrate interaction forces **F**=−∇_d_*U*, which drive atoms towards local BA coordination. The stacking arrangement cartoons use blue for boron, red for nitrogen and black for carbon. (**b**) Width of the distribution of carbon atom displacements (FWHM) as a function of the moiré pattern lattice constant at *θ*=0. The typical displacement varies from ~5 to ~8 nm when the moiré pattern lattice constant varies by a factor of four. (**c**) Vertical strains for |*ε*|=0.017 and (**d**) for |*ε*|=0.0068. Note that the vertical strain and the substrate interaction have similar spatial maps. The lower panels plot the height variation along the dashed lines of the upper panels for G- and BN-relaxed geometries. The maps for the elastic and substrate interaction energies are discussed in the Supplementary Material.

Similar to the *π*-electron Hamiltonian, the G sheet energy can be written as the sum of an isolated G layer contribution and a substrate interaction contribution that depends on the local band alignment **d**(**r**). The substrate interaction *U*(**d**) is most attractive when half the carbon atoms are directly above boron atoms, and the centres of G’s hexagonal plaquettes are directly above the nitrogen atoms (BA alignment). This alignment is energetically more stable than one in which half the carbon atoms sit on top of nitrogen (AB alignment), or one in which all carbon atoms sit on top of either boron and nitrogen atoms (AA stacking). By performing *ab initio* calculations for commensurate lattices we find that *U*_BA_<*U*_AB_<*U*_AA_. The full dependence of *U* on **d** is plotted in Fig. 2.

When *ε* or *θ* are nonzero, the substrate interaction forces plotted in Fig. 2 drive strains that attempt to match G and BN lattice constants locally and increase the sample area that is close to local BA coordination. For a given value of *ε*, the G sheet lattice constant expansion near BA points must be compensated by lattice compression elsewhere. This kind of local expansion and compression of the G lattice within the moiré unit cell was recently identified experimentally^11^,^14^.

We determine the strains by minimizing the sum of the isolated G and substrate interaction energies. For the long-period moiré lattices, the G sheet energy is accurately parameterized in terms of its elastic constants. The competition between isolated G and substrate interaction energies can then be understood by comparing the energies of the configurations in which the two terms are minimized separately. The substrate interaction energy is minimized by maintaining perfect BA alignment everywhere and therefore establishing commensurability between the BN and G lattices. Because the lattice constants of BN and G differ, this arrangement has an elastic energy cost in the G sheet. After an elementary calculation we find that the total energy per area is


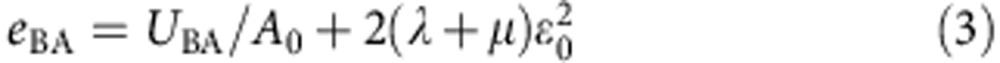


where *λ* and *μ* are elastic constants, *ε*_0_ is the relative difference between BN and G lattice constants and *A*_0_ is the unit cell area of G. The elastic energy, on the other hand, is minimized by keeping the G sheet lattice constant at its isolated value. In this configuration, because of the linear relationship between **d** and **r** the substrate interaction energy per unit area is equal to the average of *U*(**d**) over **d**:


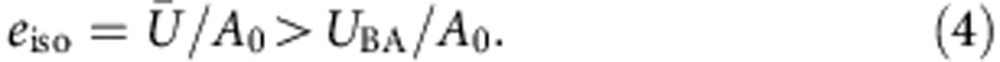


As indicated in Fig. 1, when our theoretical values for *U* are combined with the elastic constants of a G sheet, the energy of the commensurate state is substantially lower. However, equation (3) overestimates the elastic energy cost of lattice-matching between BN and G. For example, in the extreme case of a single BN layer, lattice-matching can be achieved by adjusting the lattice constants of each layer towards their mean value, approximately reducing the required strains by a factor of 2. In this case, the incommensurate structure still has lower energy, but the difference is smaller. We conclude that when they are orientationally aligned, the interaction between a G sheet and a BN sheet is nearly strong enough to favour lattice-matching.

G/BN is close enough to an incommensurate to commensurate transition that substantial strains can be driven by substrate interactions. Indeed, we find by explicit energy minimization that both vertical and horizontal strains can assume values large enough to introduce changes in the electronic structure. We determined these strains numerically for the case of a single-layer BN substrate subject to a fixed periodic potential created by the layers underneath explained in the Methods section. We find that strains in the G sheet are comparable as those in the BN layer. Note that the atomic structure, and hence the *π*-band Hamiltonian, might therefore depend on the thickness of the BN and on other features that vary from one experimental study to another. Similarly, the addition of encapsulating layers can lead to reductions in strains and hence gaps, as recently reported in ref. 11, although the gap can in principle persist.

### Strained moiré band hamiltonian

Given *H*_M_(**d**) and **d**(**r**), we obtain a sublattice-pseudospin-dependent continuum Hamiltonian with the periodicity of the moiré pattern, which is conveniently analysed using a plane-wave-expansion approach. We write the full Hamiltonian in the form


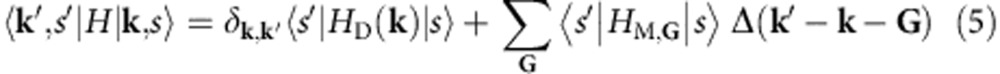


where *H*_D_ is the Dirac Hamiltonian and *H*_M,**G**_ is the Fourier transform over one period of the moiré pattern of *H*_M_(**d**(**r**)) and **G** is a moiré pattern reciprocal lattice vector. In equation (5), Δ(**k**)=1 when **k** is a moiré pattern reciprocal lattice vector and zero otherwise.

**Figure 3:**
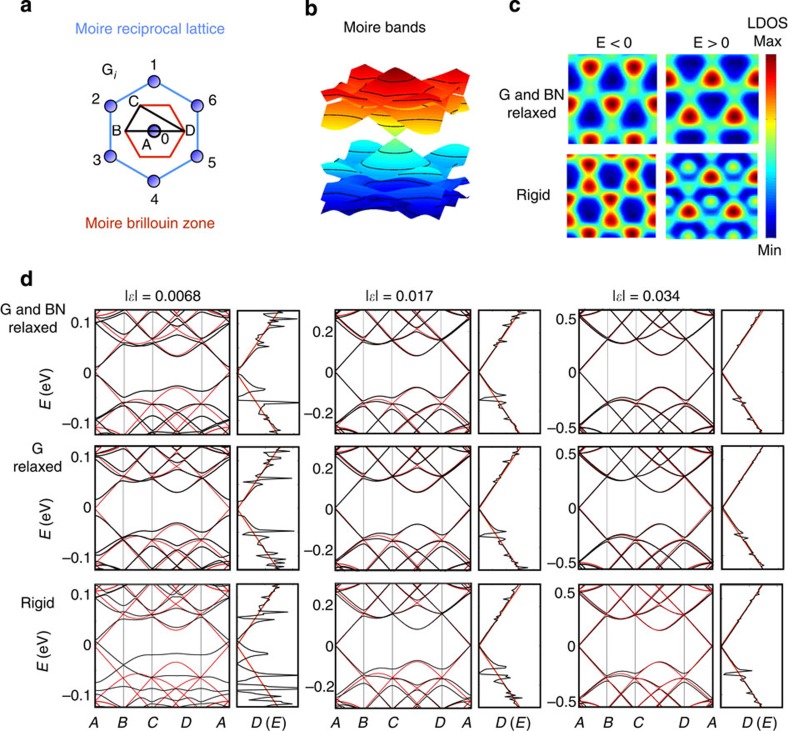
Electronic structure of G/BN heterojunctions. (**a**) Schematic representation of the moiré Brillouin zone and the moiré reciprocal lattice vectors. (**b**) Three-dimensional representation of the band structure in the moiré Brillouin zone showing superlattice Dirac point features. (**c**) Local density of state (LDOS) maps near the charge neutrality Fermi energy for G and BN relaxed and rigid lattice structures at *θ*=0 that show contrasts for electrons and holes. Lattice relaxation affects the LDOS maps. (**d**) Band structure and density of states for three different values of *ε* at *θ*=0 allowing G and BN relaxation, G-relaxation only and with no relaxation. In-plane lattice relaxation leads to sizeable band gaps in the limit of long moiré periods.

The electronic structures implied by the Hamiltonian in equation (5) for rigid lattices, for G relaxation only and for mutual G and BN relaxation are compared in Fig. 3. These results demonstrate that the electronic structure, and the gap at neutrality in particular, depends sensitively not only on *θ* and *ε* but also on the strains. Sizable band gaps appear at the neutral system Fermi level only when in-plane relaxation strains **u**(**r**) are allowed.

### Physics of the gaps

Several potential mechanisms of gap formation in neutral G have been discussed in the literature including antidots^15^, combinations of periodic scalar and vector fields^16^,^17^ and zero-line localization^18^. Our approach allows for a simple classification based on the Fourier expansion of *H*_M_. We will discuss leading contributions to the gap at neutrality in terms of the expansion of each moiré pattern Fourier component of *H*_M_ into four sublattice Pauli matrix components. We start with the **G**_0_=0 Fourier component, that is, with the spatial average of *H*_M_. In the absence of relaxation, 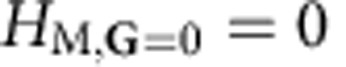 because the average of *H*_M_(**d**) is zero^9^, and **d** in this case is a linear function of **r**. (We neglect an irrelevant contribution proportional to the identity sublattice Pauli matrix *τ*^0^.) When **d** is a nonlinear function of **r**, however, the spatial average of Hamiltonian contributions that are sinusoidal functions of **d** do not vanish. Among these, the term proportional to *τ*^0^ is an irrelevant constant, and the terms proportional to *τ*^*x*^ and *τ*^*y*^, often interpreted in Dirac models as effective vector potentials, simply shift band crossings away from zero momentum. (In the continuum Dirac model of G, momentum is measured away from the Brillouin-zone corners.) However, the **G**=0 term proportional to *τ*^*z*^ produces a gap


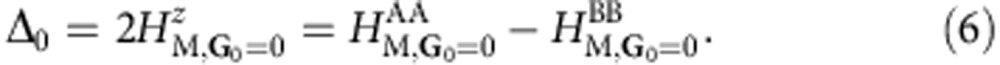


Physically this gap appears simply because the average site energy is different on different honeycomb sublattices.

The leading contributions to the gap from **G**≠0 terms in *H*_M_ are more subtle and appear at second order in perturbation theory. A perturbative treatment is in fact valid in practice because it turns out that *ℏε*|**G**| is substantially larger than *H*_M,**G**_. Applying the degenerate state perturbation theory we obtain the following expression for their contribution to the effective 2 × 2 sublattice Hamiltonian at the Dirac point:


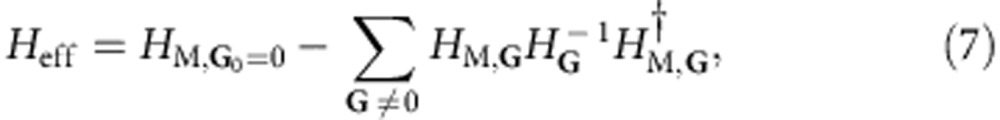


where *H*_**G**_=*ℏε***G**·*τ* (*ε* is the Dirac velocity and *τ* is the vector of Pauli matrices), ignoring the **k** dependence close to the Dirac point that will be higher order. Note that *H*_M,**G**_ connects the **k** and **k**+**G** blocks of the plane-wave-expansion moiré band Hamiltonian. Because only the term proportional to *τ*^*z*^ can produce a gap at second order, it is instructive to decompose *H*_eff_ into Pauli matrix contributions.


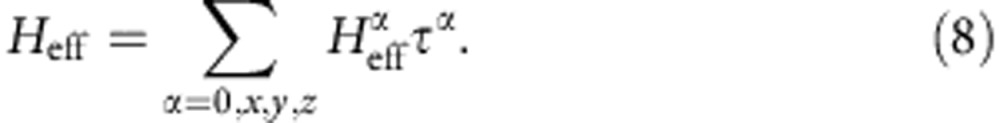


Note that higher-order terms proportional to *τ*_*x*_ and *τ*_*y*_ may in principle contribute to the gap, but we find them to be negligible. We have derived analytic expressions for 
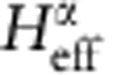
 in terms of the Pauli matrix decomposition of 
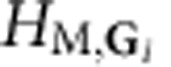
, which are discussed in detail in the Methods section. We find that, although the **G**=0 contribution to the gap is always larger, the **G**≠0 contributions are not negligible. Both the difference in the spatial average of sub-band energies and the detailed form of the full substrate interaction Hamiltonian play a role in determining the size of the gap at neutrality, and both are sensitive to the detailed structure of the lattice relaxation strains.

In G, nonlocal exchange interactions are expected to enhance gaps^19^,^20^,^21^ at neutrality produced by sublattice-dependent potentials. We have performed plane-wave-expansion self-consistent Hartree–Fock calculations in which Coulomb interactions are added to the moiré band Hamiltonian we have discussed. The calculations were performed using effective dielectric constants bracketing the expected values between *ε*_r_=2.5 and *ε*_r_=4. When all effects are included we find band gaps ~20 meV, as shown in the inset of Fig. 1. The values chosen for *ε*_r_ partly account for dielectric screening by the substrate and partly accounts for dynamic screening effects in the same spirit as in the screened exchange functionals used in density functional theory. We have previously used a similar dielectric constant of *ε*_r_=4 to successfully predict spontaneous band gaps ~50 meV in ABC trilayer G^22^. Further details of the Hartree–Fock theory in moire superlattice bands will be presented elsewhere.

## Discussion

We have derived a *π*-band continuum model Hamiltonian intended to describe states near the Fermi level of G/BN and used it to address the energy gaps often observed in neutral G when it is nearly aligned with a BN substrate. In this theory the interaction of *π*-band electrons with the substrate is described by a local but sublattice-dependent term *H*_M_ that is dependent on the local relative displacement of the G sheet and substrate honeycomb lattices, **d**(**r**). When neither the G sheet’s carbon atoms nor the boron and nitrogen atoms in the substrate are allowed to relax, **d**(**r**) is a linear function of position because of the difference between the lattice constants *ε* and because of difference in orientations specified by a relative angle *θ*. The gap produced by substrate interactions in the absence of relaxation reaches its maximum at *θ*=0, but is never larger than a few meV and too small to explain experimental observations. Only by allowing the carbon and substrate atoms to relax we can explain the much larger experimental gaps.

The moiré pattern formed by G and a BN substrate is characterized in the first place by the lattice constant difference *ε* and by the relative orientation angle *θ*. These two quantities can be changed only by collective motion of many atoms. We take the view that because of large barriers and weak thermodynamic drivers these two macroscopic variables are not in practice relaxed to equilibrium values. We therefore view them as observables that characterize particular G/BN systems and calculate relaxation strains and π-band electronic structure as a function of *ε* and *θ*, and hence as a function of the moiré pattern period. The explicit calculations reported on in this paper are for *θ*=0, the orientation that leads to large experimental gaps.

To account for relaxation strains, we minimize the total energy with respect to carbon and substrate atom positions. For this purpose we assume that the interaction energy *U* between G and substrate is also a local function of **d** and obtain *U*(**d**) from density functional calculations of commensurate structures. The strains minimize the total energy by increasing the number of carbon atoms that are on top of boron atoms and the number of hexagonal carbon atom plaquettes that are centred above nitrogen atoms. Our study emphasizes that atom relaxation in the BN sheets is as important for the electronic structure as atom relaxation in the G sheet. Although only atom positions in the top BN sheet are important for electronic structure, these will be affected by interactions with atoms in remote layers. We have performed calculations for two extreme cases, rigid BN atoms and a single layer of BN in which atom positions relax to minimize total energy, finding that relaxation increases the energy gap substantially. The physical origin of these gaps can be revealed by expanding the continuum model *π*-band Hamiltonian in terms of Pauli matrix pseudospin operators and in terms of moiré pattern reciprocal lattice vector components. Because of the wide *π*-band width and the relatively short moiré periods, the contribution of each term in the Hamiltonian to the gap can be analysed using the leading order perturbation theory. The **G**≠0 terms that capture detailed spatial patterns contribute at second order and are not negligible. The largest contribution to the gap comes from the **G**=0 term, which vanishes in the absence of lattice relaxation and has a very simple interpretation. Because of relaxation strains the average site energy in the carbon sheet is different for the two carbon atom sublattices. It is well known that this type of perturbation produces a gap at the Fermi level of a neutral G sheet. Surprisingly, the gap is a substantial fraction of the gap of the same origin present in the commensurate BA-aligned G on BN. The gaps are therefore due to the contrast between the local classical physics of energy minimization with respect to atom position, and the wide π-bands and nonlocal quantum physics, which forces the quantum wave functions to be smooth and sensitive mainly to spatial averages over the moiré period. When many-body interaction effects^19^ are accounted for, these gaps are enhanced to values that are consistent with experiment. The approach described in this paper can be applied to other van der Waals materials that can form heterojunctions in which different layers have slightly different lattice constants or differ in orientation—such as transition metal dichalcogenide stacks or twisted bilayer G^9^,^23^,^24^,^25^,^26^,^27^. Finally, we note that after the submission of this work, two recent papers also appreciated the importance of strain in the moiré superlattices formed in van der Waals heterostructures^28^,^29^.

## Methods

### Elasticity theory and interaction potentials

The elastic energy functional was modelled using the Born-von Karman plate theory^30^. Neglecting the small bending rigidity of G *κ*=1.6 eV (ref. 31) the elasticity theory depends on the two Lamé parameters whose estimates for G from empirical potentials gives *λ*=3.25 eV Å^−2^ and *μ*=9.57 eV Å^−1^ (ref. 32) in the low temperature limit, and for a single BN sheet we have used *λ*~3.5 eV Å^−2^ and *μ*~7.8 eV* *Å^−2^ obtained averaging the local density approximation (LDA) and generalized gradient approximation (GGA) values, respectively^33^. The potential energy has been parametrized from the stacking-dependent and separation-dependent energy curves in ref. 33 calculated at the exact exchange+random phase approximation (EXX+RPA) level as we show below.

The total elastic energy superlattice area is given by^30^,^34^,^35^


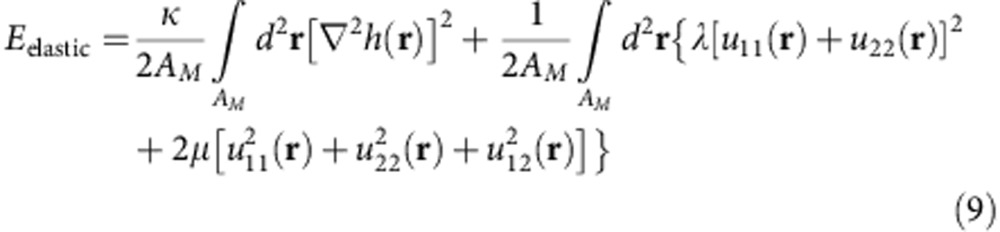


The strain tensors *u*_*ij*_(**r**) associated with the deformation of the G layer depend both on the in-plane displacements and heights in Monge’s representation:


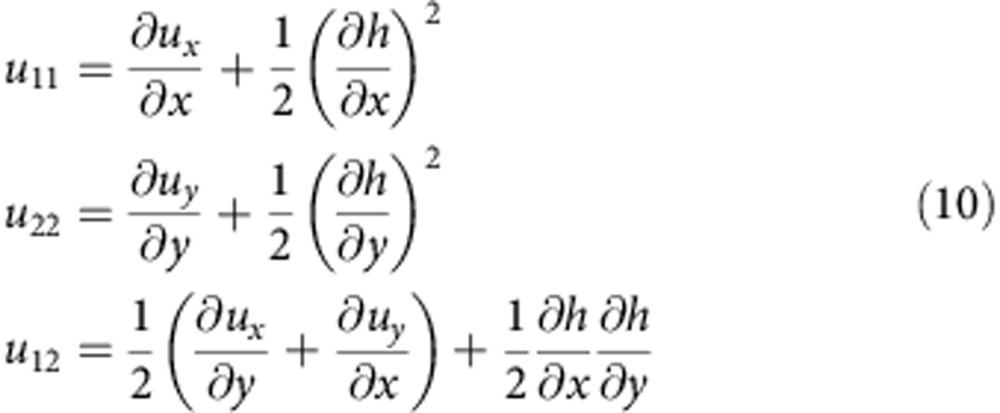


In a practical calculation, it is convenient to use an integration domain that remains fixed for every moiré period. For this purpose we use rescaled coordinates to operate in the coordination vector **d** defined in the unit cell of G. Using the chain rule to relate the reduced vector **d** in G’s unit cell and the real-space **r** coordinates for zero twist angle and assuming variable lattice constant mismatch *ε* we have


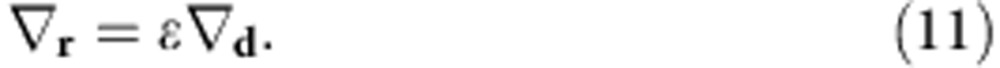


When we neglect the contributions from the height variation the elastic energy can be written as


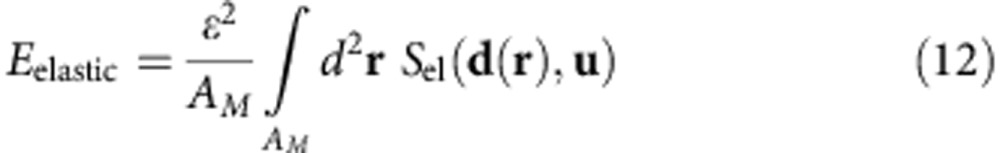


where *S*_el_ represents the integrand of equation (12) in rescaled coordinates **d**. This form shows more explicitly a *ε*^2^ weakening of the elastic energy as the lattice constant mismatch becomes smaller.

### Parametrization of the hamiltonian

The diagonal and off-diagonal elements of the Hamiltonian for a fixed interlayer separation distance can be written in the sublattice basis in a manner similar to the pseudospin representation used in ref. 9,


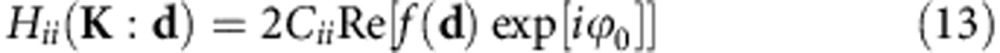



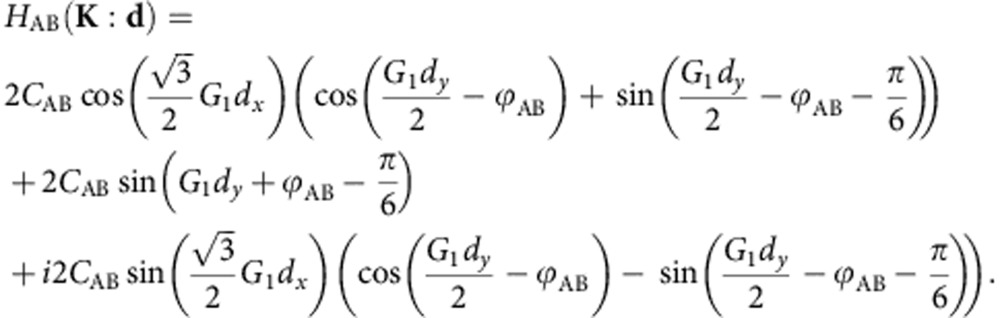


The out-of-plane *z* axis layer separation dependence can be incorporated into the three main coefficients *C*_*ii*_(*z*) with an exponentially decaying behaviour in the form


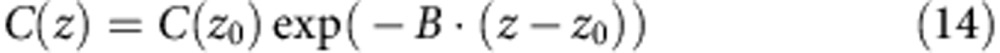


where *z*_0_=3.35 *Å*, and the three decay coefficients *B*=3.0, 3.2, 3.3 *Å*^−1^ for each one of the terms of the Hamiltonian in the sublattice basis were found fitting the *z* dependence between 2.8 and 5 Å, where we use the parameters obtained from *ab initio* calculations


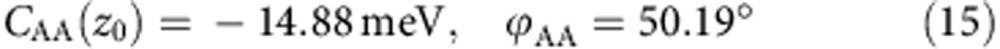



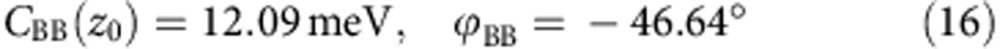



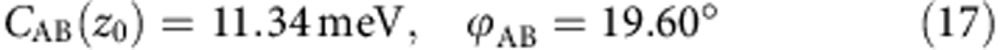


whose equivalent values in the pseudospin basis had been calculated previously^9^. The variation of the phase with *z* shows a weak linear dependence and we can approximate it as a constant value. The effects due to lattice relaxation can be conveniently incorporated when calculating the Fourier expansion of the above Hamiltonian by accounting for the in-plane displacement **u**(**r**)=(*u*_*x*_(**r**), *u*_*y*_(**r**)) in the stacking coordination vector **d**(**r**)=**d**_0_(**r**)+**u**(**r**), and the height *z*=*h*(**r**) that represents the local distance of G to BN, where **r**=(*x*, *y*) is a two-dimensional (2D) vector. Both the displacement vectors **u**(**r**) and the height maps *h*(**r**) are assumed to respect the moiré periodicity and are therefore modelled from the scalar fields that we introduce in the following.

### Moire pattern scalar functions and vector fields

The scalar functions used to obtain the moiré superlattice pattern for the height and the displacement vectors from their gradients have used Φ written as a Fourier expansion in **G** vectors as


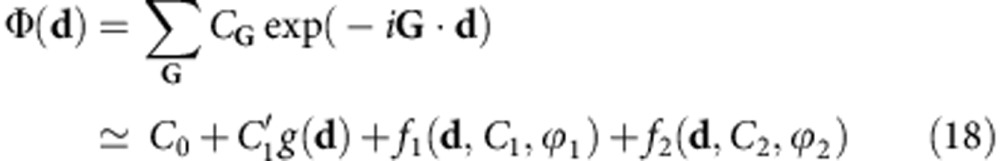


where *C*_**G**_ is in general a complex number, and we retain up to three nearest **G** vectors for the scalar field that preserves the symmetry of triangular lattices. The parameters *C*_0_, 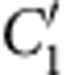, *C*_1_, 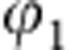, *C*_2_ and 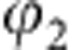 are real valued constants and we defined auxiliary functions *f* and *g* in terms of the triangular lattice structure factors similar as those used in a general tight-binding model of G^36^. The Fourier expansion coefficients within the first shell consisting of 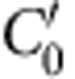 and the first shell 
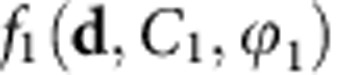
 are often good representation of the solutions that vary smoothly in real space and respect the symmetry of the triangular superlattice. For brevity in notation, here we use (*x*, *y*) to indicate the (d_*x*_, d_*y*_). The *f* function


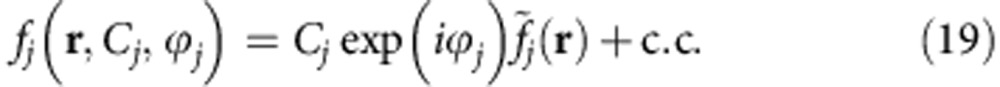


is defined in terms of the structure factors


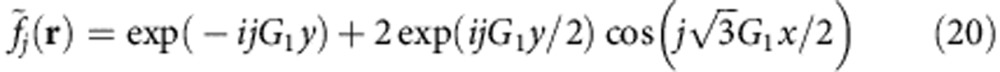


where *G*_1_=4π/3*a*, where *a* is the real-space periodicity of the moiré superlattice and *j*=1, 2. These are momentum space analogues of the real space intersublattice hopping structure factors in a honeycomb lattice^37^. The explicit form of the functions defined along the symmetry lines *x*=0 or *y*=0 can be obtained from sums of


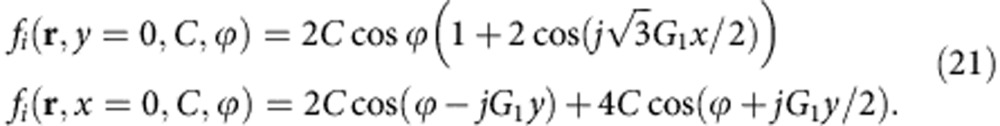


The analytical expression for the *g* function shell contribution reduces to a simpler form


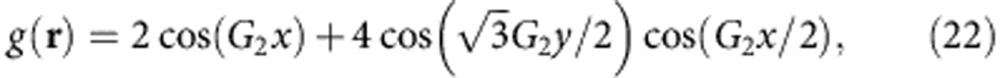


where *G*_2_=4*π*, that for the symmetry lines reduces to


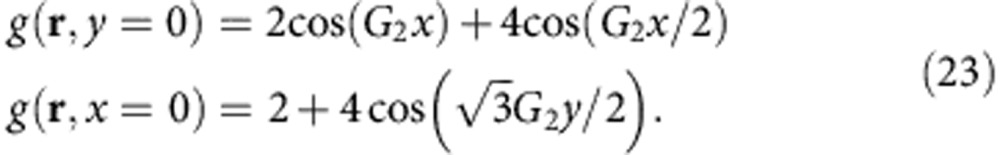


The vector fields such as in-plane forces, displacement vectors and stresses can be obtained as gradients of the scalar potentials given by the above forms that can preserve the symmetry of the triangular moiré superlattice. The vector field that can be obtained from the gradient of the scalar field is


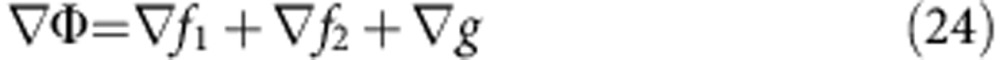


and can be obtained taking the respective partial derivatives. Thus, we have


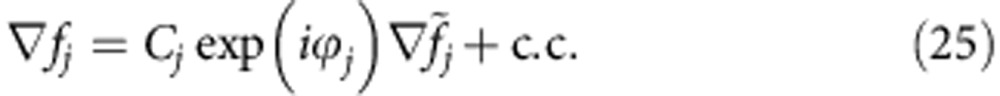


where the partial derivatives of the constituent functions are given by


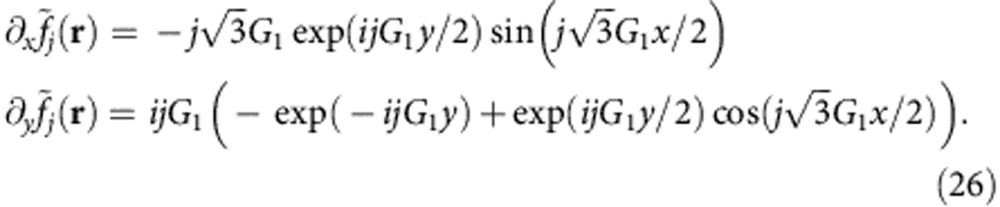


For the *g* terms we have


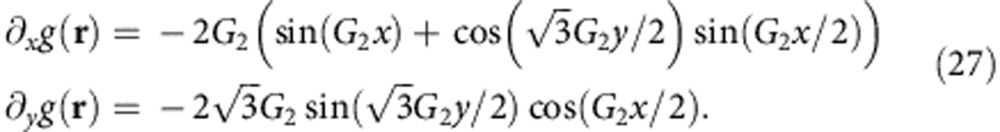


**Figure 4:**
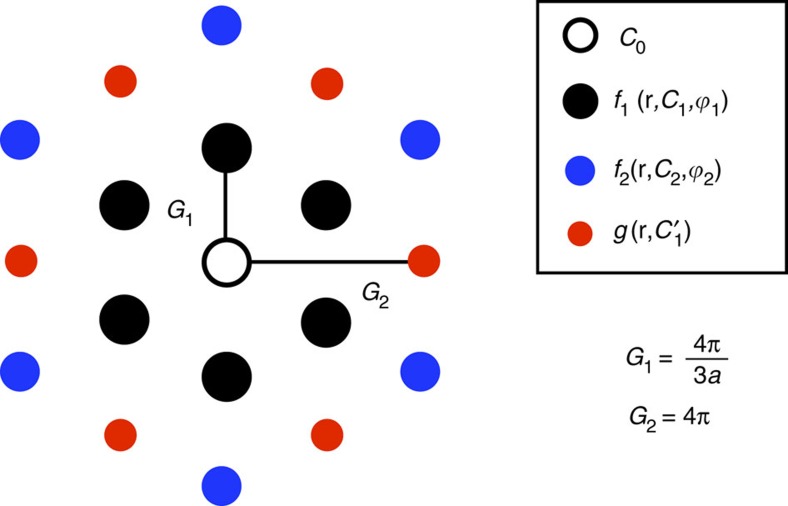
Variational space associated with the first three G-vector shells. Representation of the different **G**-vector shells corresponding to the structure factors *f*_1_, *f*_2_ and *g* and the parameters that define the specific form of the scalar functions.

Likewise, higher-order derivatives used in the stress tensors or the gauge fields can be evaluated analytically. The pair of parameters *C* and 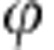 for each *f*_*j*_ function and the single parameter accompanying the *g* function specify the variational space we used to minimize the energy functionals. Because the *f*_1_ term captures the first harmonic contribution, the different variables such as **u**, *h*, *E*_potential_ can be characterized in terms of just two parameters *C*_1_ and 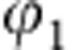, or up to three when the average value of the origin *C*_0_ is required. The most relevant **G**-vector Fourier components used in our calculation corresponding to *f*_1_, *f*_2_ and g functions are represented in Fig. 4. The self-consistent Hartree–Fock calculations used effective relative dielectric constants between *ε*_*r*_=2.5 and 4 and 217 *k*-points in the moiré Brillouin zone.

### Parametrization of the potential energy

Likewise, it is convenient to use the parametrization of the potential energy in the coordination vector **d**(**r**) and the interlayer separation height. The potential energy term has been parametrized from EXX+RPA calculations, binding energy curves for different stacking configurations^33^ as a starting point to extract the potential energy curves needed for the formulation of the Frenkel–Kontorova (FK) model for this 2D bipartite lattice. We can neglect the van der Waals tail corrections from the bulk that bring the equilibrium distances closer because their influence in distinguishing different stacking energies are small. We make use of the property that the energy landscape for a fixed *z* axis separation is given by a simple expansion in the first shell of G vectors in Fourier space^9^ to represent the energy map with three parameters. As noted previously, the simplest approximation for a scalar field that varies smoothly in real space with the triangular lattice symmetry is given by


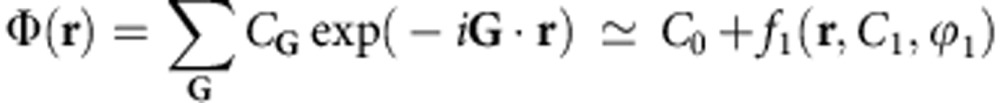


where the constants *C*_**G**_ are complex numbers. Owing to this simple form it is possible to parametrize the whole energy landscape from the values of the potentials at three inequivalent stacking configurations, for example, the three symmetric stacking configurations AA, AB and BA. Its explicit expression


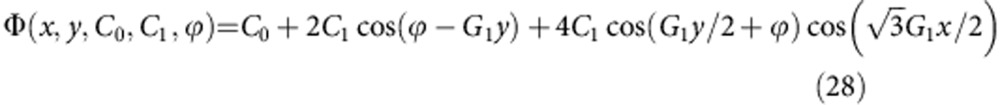


repeats with the periodicity of a triangular lattice. The scalar function at the three distinct symmetry points in units of G’s lattice constant


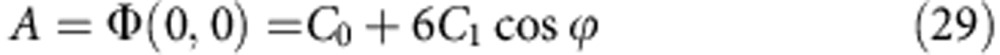



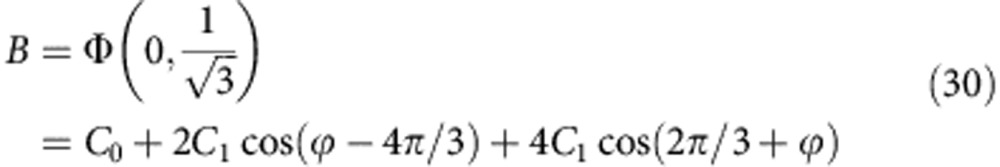



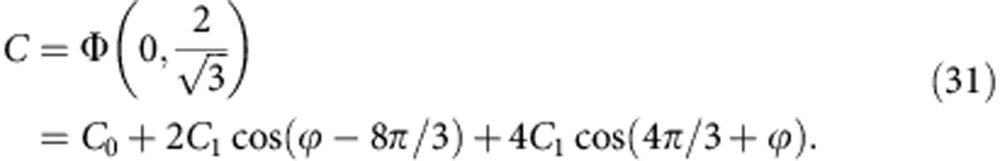


These equations lead to the explicit values of the parameters


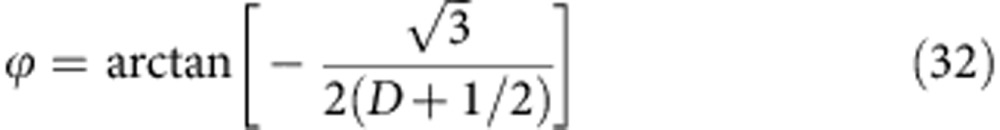



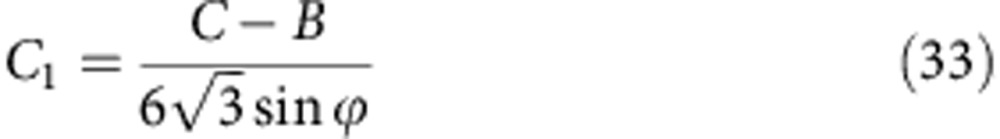



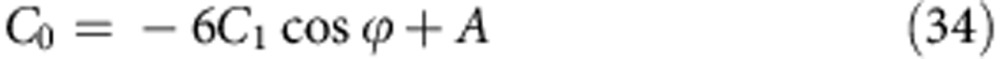


where we have used the relation *D*=(*A*–*B*)/(*B*–*C*). From the layer separation *z* dependence of these three coefficients *C*_0_(*z*), *C*_1_(*z*) and 
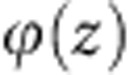
 we can obtain the complete potential landscape *U*(*x*,*y*,*z*) that we need for our model. We note that the *C*_0_(*z*)=(*A*(*z*)+*B*(*z*)+*C*(*z*))/3 term is the average value of Φ(*x*, *y*) in the periodic domain for every value of *z* and that the remaining *C*_1_(*z*) and 
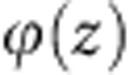
 terms accounts for the landscape of the energy in the first harmonic approximation, which is often an accurate approximation for functions varying smoothly with the moiré pattern^9^. The difference between this average and the minimum *U*_dif_=*U*_av_−*U*_min_ gives a measure of the in-plane forces associated with the energy gradient in a FK problem^37^. The numerical values for *A*(*z*), *B*(*z*) and *C*(*z*) for the binding energy curves as a function of separation distance *z* can be obtained from the calculations provided in ref. 33. They can be interpolated numerically or, alternatively, we can use analytic fitting expressions similar to that shown in ref. 38 used in the G/G case. We define the auxiliary functions


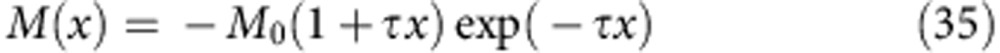



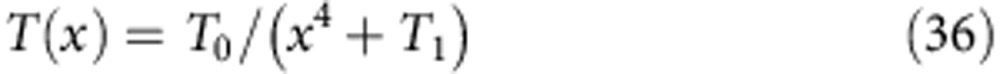



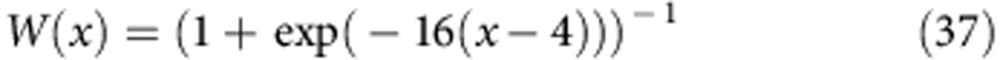


with the parameters *M*_0_=0.06975, *τ*=7, *D*_0_=3.46, *T*_0_=−10.44, *T*_1_=−58.87 to define the fitting function for the average value of *C*_0_(*z*) for all the stacking configurations through


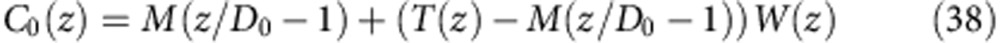


where *z* is given in angstroms. We used a rather simple model for *W*(*x*), which is fairly accurate but can still be improved through additional parameters to better capture the behaviour away from the equilibrium point. The *z* dependence of the *C*_1_(*z*) term is easily captured through an exponentially decaying form


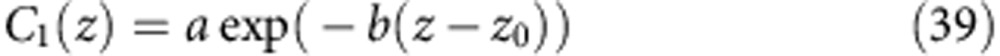


where *a*=2.226, *b*=3.295 and *z*_0_=1.295, *a*_0_=2.46 Å is the lattice constant of G. The 
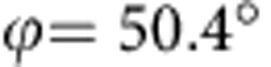
 term shows a weak linear dependence with respect to *z*; therefore, we use a constant value. When necessary, the long-ranged van der Waals tails originating from the bulk BN layers can be added through


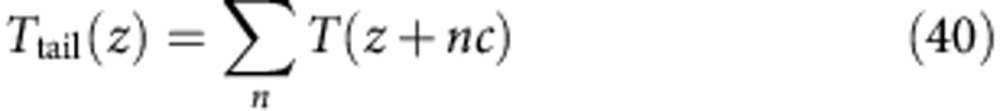


where *c* is the separation lattice constant between the layers and whose sum saturates quickly. However, this correction term has a small influence for the differences in energy for different stacking arrangements and we neglect this term. The energy landscape plots for a fixed separation distance *z*_0_=3.4 Å presented in Fig. 5 allow to estimate the average in-plane traction force being applied on the two-inequivalent carbon atoms in the unit cell. Even though the LDA-binding energies are substantially smaller than that shown in an EXX+RPA calculation, we find that this in-plane energy map obtained through parametrization in Fig. 2 is closely similar to the LDA energy map obtained in ref. 9, whose agreement is attributable to the dominance of short-range character of the interactions near equilibrium distances that is captured reasonably well by the LDA approximation^38^.

From this potential landscape per two carbon atom unit cell we can infer the potential experienced by the individual carbon atoms that can be useful for lattice force-field calculations where the higher-energy optical modes are treated explicitly. This is performed assuming that the total potential energy consists of the sum of the potentials experienced by each carbon atom, which is separated by a distance 
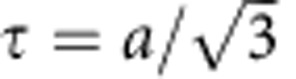



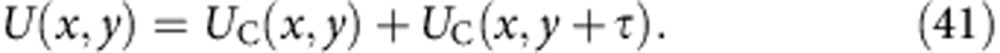


Solving the above equation we ge


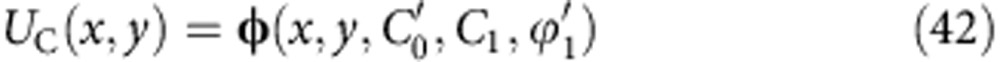


where 
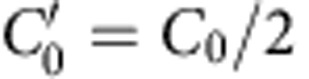
 and 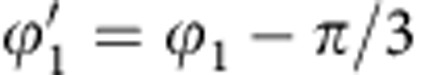. Likewise, if the long-range van der Waals tails are used they would need to be reduced to one half of its value. In Fig. 5b we show the potential energy repeated over several periods as well as the energy landscape seen by each carbon atom, derived assuming additivity in the total potential energy.

### G relaxation-only model

The resolution of the elastostatic problem of G subject to a superlattice potential requires the minimization of the total energy functional


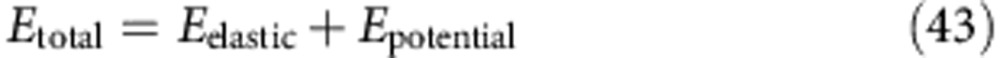


where *E*_elastic_ is given in equation (12) and the potential energy is given by the integral in the moire supercell of area *A*_M_ of the potential energy kernel *U*(**r**, **d**, *h*) given in equation (28)


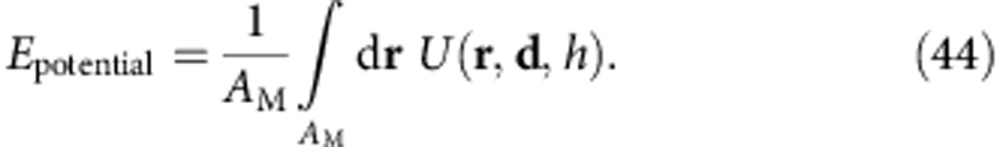


**Figure 6:**
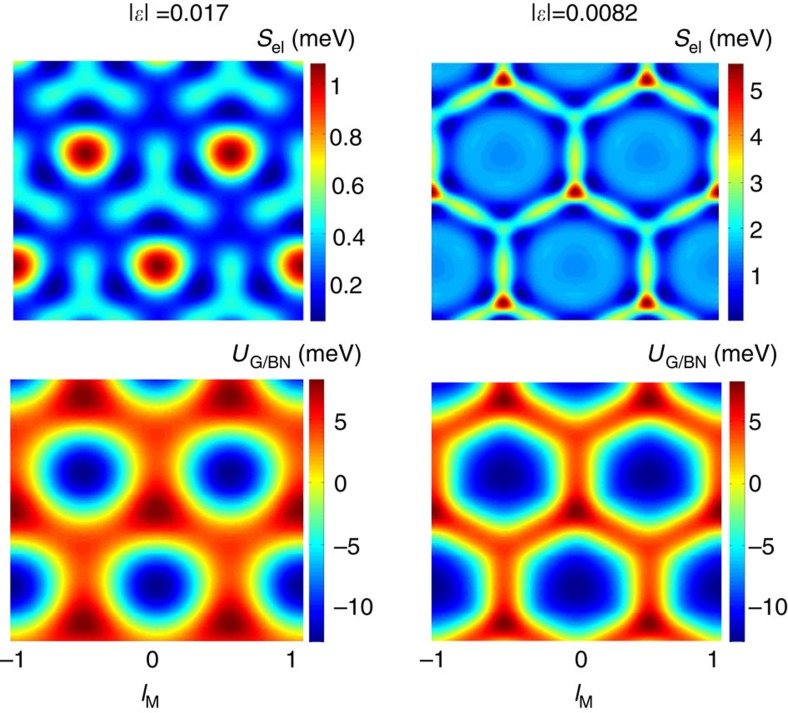
Elastic and potential energy maps for relaxed geometries. Map of local elastic and potential energies per unit cell area (see equations 12, 28) corresponding to small and large strains using constant *h* model corresponding to lattice constant differences of |*ε*|=0.017 and 0.0082, repectively. The large strain configuration we represent here is just before the point of steep transition, which happens for longer moiré periods than when the *z* axis relaxation is allowed.

The stacking coordination vector is modelled as


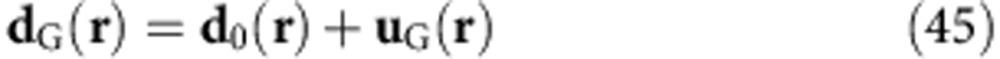


assuming that the substrate produces a rigid periodic potential pattern. We use the gradients of the scalar field in equation (24) to model the displacement vectors **u**_G_. The local elastic and potential energy maps corresponding to the small and large strain limits are represented in Fig. 6.

### Coupled relaxation of the BN lattice

**Figure 7:**
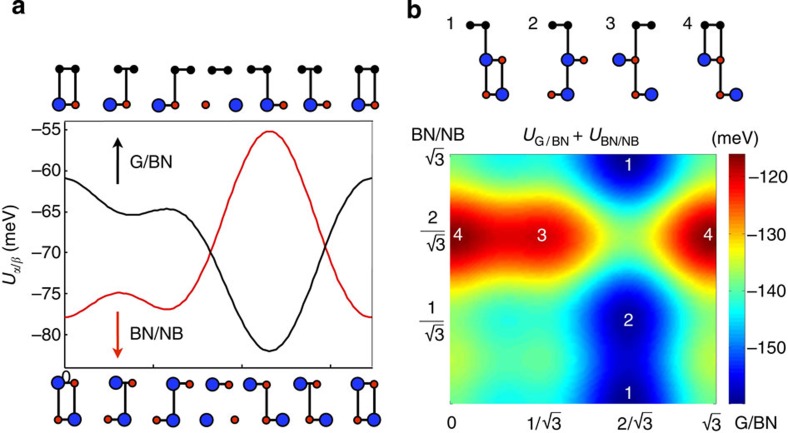
Coupled G and hBN interaction energy map. (**a**) We show the total energy per unit cell area for sliding along the vertical *y* axis for different stacking configurations for G/BN within RPA and BN/NB heterojunctions within LDA near the equilibrium interlayer separation for fixed *c*=3.4 Å. The energy curves were obtained using the information at three different symmetric stacking configurations for AA, AB and BA for interlayer sliding vectors *τ*_AA_=(0, 0), 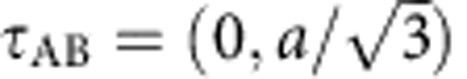 and 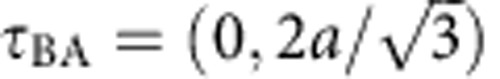. The energy minimum for G/BN stacking happens at *τ*_BA_, whereas for BN/NB the energies are smallest near *τ*_AA_ and *τ*_AB_. (**b**) We show the stacking configurations for G/BN and BN/NB and minimize the total energy, which shows that deformation of the topmost BN layer is easier when it preserves the BA stacking of the G/BN hererojunction. The minimum energy configurations are indicated with labels 1 and 2, whereas the maximum energy ones are labelled with 3 and 4.

**Figure 8:**
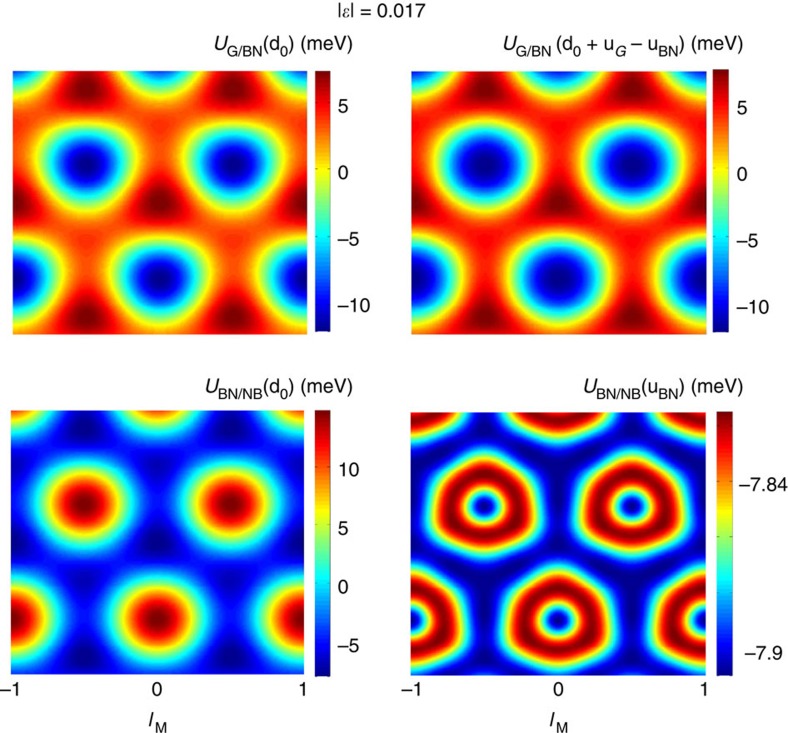
Interlayer potential energy maps. The potential energy maps *U*_G/BN_ and *U*_BN/NB_ are represented in real space. The left column represents the interaction potentials of rigid G and BN sheets. The right column shows the potential energy maps for *U*_G/BN_ and *U*_BN/NB_ corresponding to the relaxed geometry configurations determined by the strains in the G and boron nitride sheet given by u_G_ and u_BN_, respectively.

**Figure 9:**
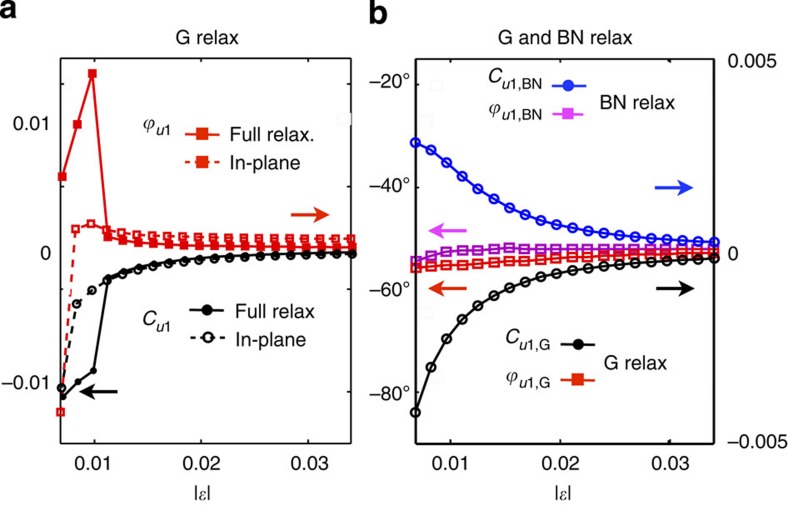
Solution strains of the elastostatic problem. (**a**) Elastostatic solutions for the strains in the G sheet relaxation only that is subject to the potential of a rigid BN substrate. For smaller |*ε*|, the potential energy dominates and the deformation becomes larger. The increase in the deformation is steady until it reaches a tipping point where the solutions become unstable. Comparison of in-plane relaxation only and that allowing out-of-plane relaxation shows that the both approximations give similar in-plane displacements, but allowing the full relaxation makes the transition easier. (**b**) Elastostatic solutions of the coupled G and topmost BN layer subject to the potentials of a rigid BN layer potential underneath. We notice that the magnitude of the in-plane deformation of the G and BN sheets are comparable to the strains in the BN sheet as the latter can relax along the easy sliding axis directions.

**Figure 10:**
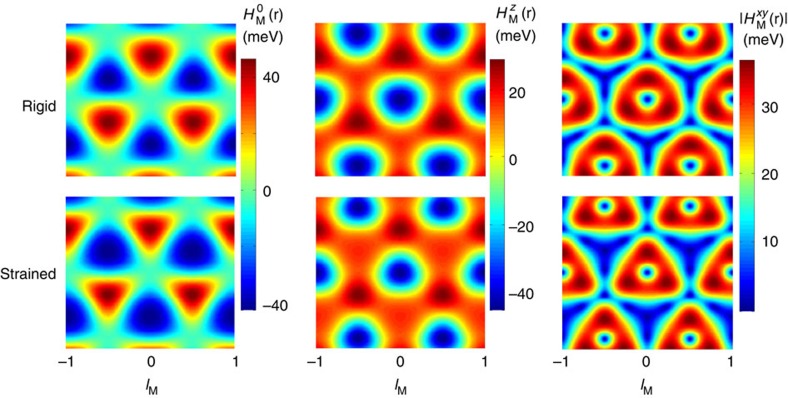
Real-space representation of the pseudospin Hamiltonian. We considered the unrelaxed (top row) and constant *h* in-plane relaxed (bottom row) geometries near zero twist angle. The 
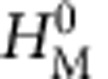
 term accounts for the site potential fluctuations normally seen in scanning probe studies, the 
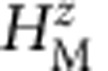
 term is the mass term dictating the local band gap in real space and 
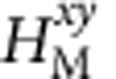
 reflects the anisotropic strains.

Here we explore the influence in the elastic of energy of G when the BN atoms of the topmost layer in the substrate are allowed to relax in response to the stacking rearrangement of the G sheet. The coupled motion of the substrate atoms contributes in decreasing the total elastic energy of the G BN heterojunction because a smaller displacement in the G sheet is needed than if the substrate remains rigid. For solving the coupled G/BN elasticity problem we will assume that the topmost BN sheet is subject to a potential stemming from the G sheet itself and the BN layers underneath, assuming that the BN atoms below the topmost layer remain fixed. The potential energy for fixed interlayer separation of *c*=3.4 Å for G/BN and BN/NB along the *y* direction of stacking arrangement vector is shown in Fig. 7. A comparison of the potential energy maps and the magnitudes of the moire strains are illustrated in Figs 8 and 9, respectively. Their influence in the real-space pseudospin Hamiltonian map is shown in Fig. 10 and the magnitudes of the pseudomagnetic vector potentials resulting from strains are shown in Supplementary Fig. 1. The interaction potential between the two topmost BN layers are defined by *C*_1_=−2.47 meV and 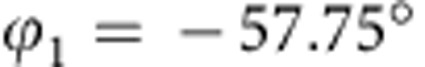 through the funciton 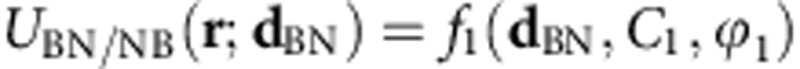.

Even though the binding energies and forces predicted by the LDA typically underestimate the values obtained from higher-level RPA calculations^39^,^40^,^41^,^42^, we assume that the energy landscape for different strackings We used LDA energies for BN/NB coupling as a function of sliding, assuming that their sliding energy maps are comparable to EXX+RPA as we found for the G/BN case. The total energy of G/BN/NB where both sheets are allowed to relax is given by the sum of the elastic and potential energies of G and the topmost BN sheet. The total potential energy term can be obtained from the interaction energies between the neighbouring layers through


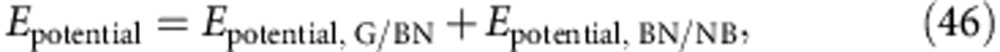


and can be calculated from the parametrized potential energies evaluating the integrals in the moiré supercell


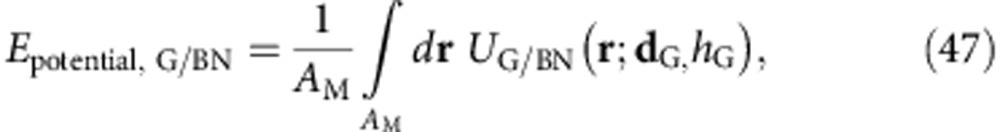



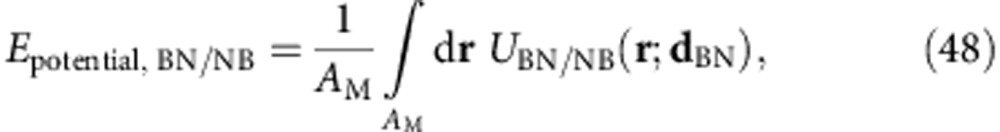


where the kernels are functionals of the local stacking coordination functions **d**_G_ and **d**_BN_ that depend on the displacements relative to the neighbouring layers


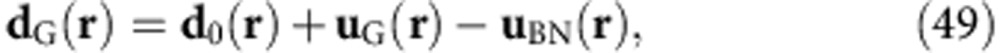



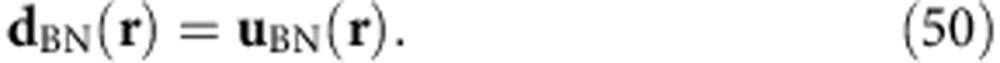


We used explicit labels G/BN and BN/NB to distinguish the interaction potentials. For the G sheet, the only relevant reference frame is the topmost BN layer, whereas the latter interacts both with the G sheet and the BN layers underneath whose coordinates are assumed to remain fixed. Further discussions on the equilibrium strains and the Fourier components of the Hamiltonian can be found, respectively, in Supplementary Note 1 and Supplementary Note 2.

### Second-order perturbation theory

Further insight on the contributions to the band gaps can be achieved from second-order perturbation theory from the first shell approximation^38^,^39^,^40^,^41^,^42^. We distinguish two scenarios: one for rigid unrelaxed lattices and another where strains are allowed to modify the stacking coordination. Formally it is possible to show that for rigid unrelaxed lattices the in-plane 
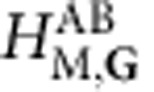
 gives a zero contribution to the band gap to second order in the perturbation theory. When in-plane strains are allowed, band gaps develop owing primarily to a nonzero average mass, and all three pseudospin components make a nonzero contribution to the gap to second order. Among these, in our calculations the in-plane pseudospin terms contribute to the gap with a smaller magnitude than the Fourier expansion of the mass terms 
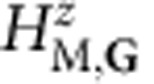
.

First, for unrelaxed configuration the effective 2 × 2 Hamiltonian is obtained from the perturbation theory around the Dirac point. Our initial Hamiltonian is a 2*N* × 2*N* matrix, where *N* is two times the number of Moiré reciprocal lattice vectors in the Fourier transform. Treating the 2 × 2 diagonal blocks as the unperturbed Hamiltonian, the second-order degenerate perturbation theory gives an effective Hamiltonian for the low-energy states,


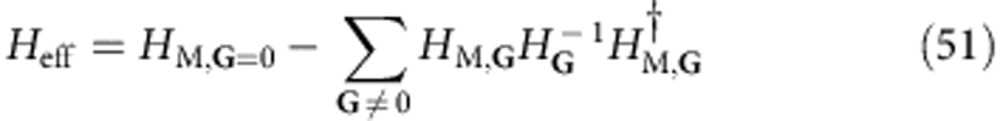


where *H*_**G**_ are the 2 × 2 blocks in the Hamiltonian associated with the moiré vector **G**, and *H*_M,**G**_ connect the **k** and **k**+**G** blocks of the Hamiltonian. If we ignore for a moment the relaxation because of in-plane strains, the diagonal blocks are 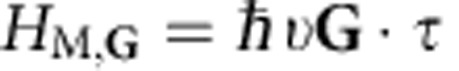, which has an inverse 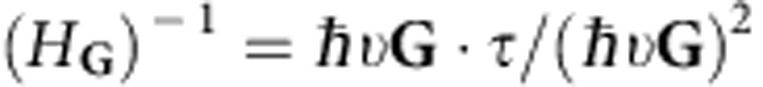. We can decompose both the effective Hamiltonian and the *H*_M,**G**_ into terms proportional to Pauli matrices,


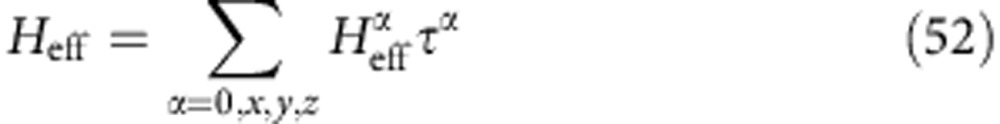



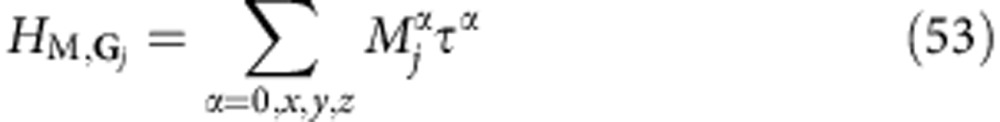


Since *H*_eff_ is hermitian, the parameters 
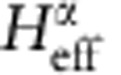
 must be real numbers. However, each block 
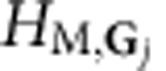
 is not necessarily hermitian; therefore, 
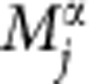
 are complex numbers. Plugging in the decomposed forms, and restricting to just the nearest shell of reciprocal lattice vectors *j*=1,…,6 (we use the index *j*=0 for **G**=0), we get


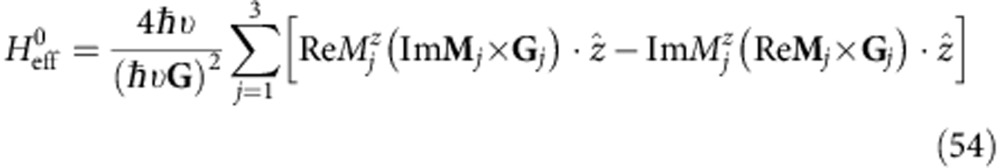



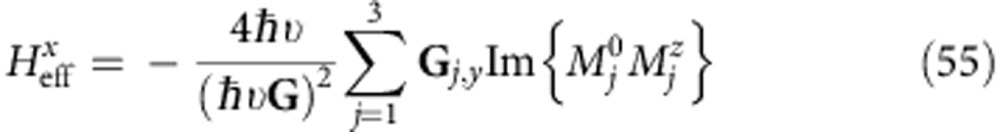



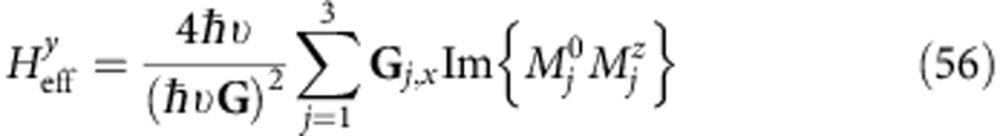



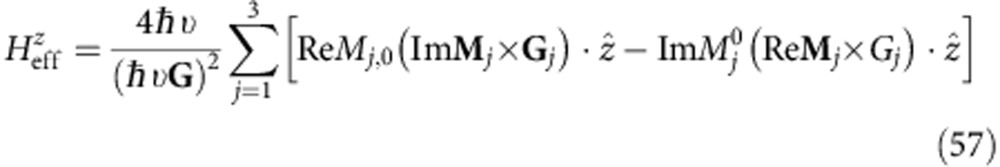


The sums in the above equations are restricted to *j*=1, 2, 3 because of the relation **G**_*j*+3_=−**G**_*j*_ and the property of the corresponding matrices, 
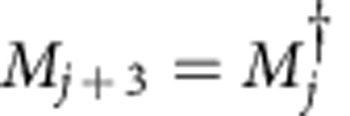
. We will now prove that *h*_*x*_=*h*_*y*_=0. The moiré Hamiltonian has the property 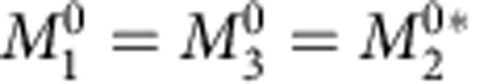 and 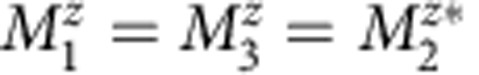. Therefore, 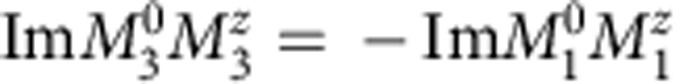. However, **G**_1_−**G**_2_+**G**_3_=0. Examining the above equations, we see that 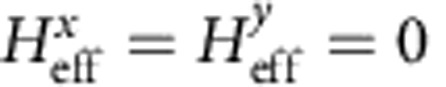 because of the symmetry properties of the Hamiltonian, and the gap for the unrelaxed configuration arises entirely from a mass term 
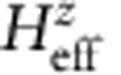
.

We have numerically calculated the low-energy eigenvalues as a function of the parameters 
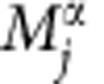
 to verify the second-order perturbation theory result and show which terms contribute at third order and higher. Our numerical calculations are performed by multiplying each of the 
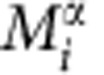
 by interpolation parameters *λ*_α_, which range from 0 to 1, thus keeping the same relationship (magnitude and phase) between the different **G**_*i*_ terms while allowing us to see explicitly the power law behaviour of the gap due to each term.

First, we set all *λ*_α_=0 except for one. Power law fits show that there is no second-order contribution from any of the terms individually, see Supplementary Fig. 2. The *λ*_*z*_≠0 term contributes at the third order, while all others are fifth order or higher.

Next, we look at the interplay between the different matrix elements 
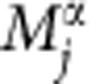
, which are found to contribute to the perturbation theory results for 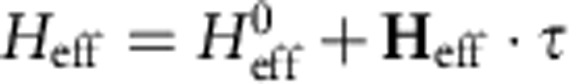. The aforementioned Supplementary Fig. 2 and the breakdown of the different contributions to the band gap shown in Supplementary Fig. 3 confirm that to second order, the gap is not opened by terms proportional to *M*^*x*^*M*^*z*^, *M*^*y*^*M*^*z*^, *M*^0^*M*^*z*^ and *M*^*x*^*M*^*y*^. The first two, *M*^*x*^*M*^*z*^ and *M*^*y*^*M*^*z*^ do contribute to the energy levels at second order: they lead to a nonzero 
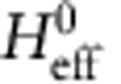
, which does not open a gap. The *M*^0^*M*^*z*^ term we found to be zero in the second-order perturbation theory due to the symmetry of the Hamiltonian, which is verified here. Finally, the term *M*^*x*^*M*^*y*^ does not appear in the second-order perturbation theory at all, which is again confirmed by our numerical results. The only terms that contribute to the gap at the second order, and are therefore most efficient at opening a gap, are *M*^0^*M*^*x*^ and *M*^0^*M*^*y*^.

For relaxed configuration there is a large effect on the size of the gap. This is due primarily to the emergence of nonzero mass in the 2 × 2 block 
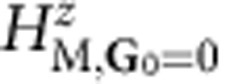
. This term alone slightly overestimates the gap. We again calculate a second-order perturbation theory, equation (51). The main effect is a zero-order contribution to the gap, 
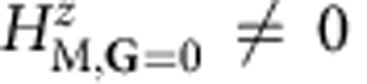
, equation (61) below. Assuming a form 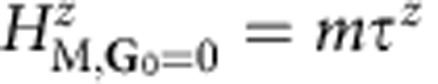, the perturbation theory restricted to the six nearest reciprocal lattice vectors gives the following equations,


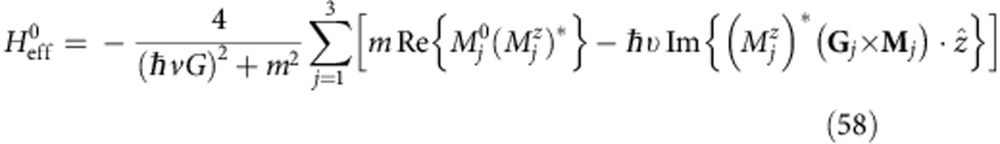



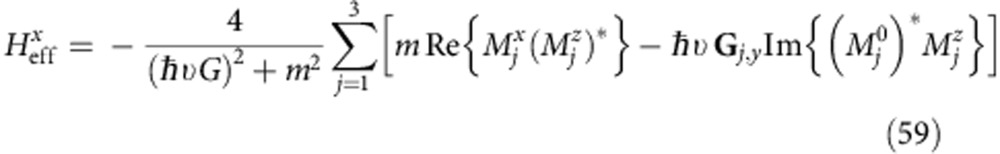



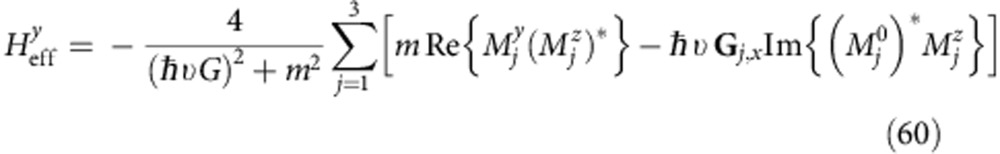



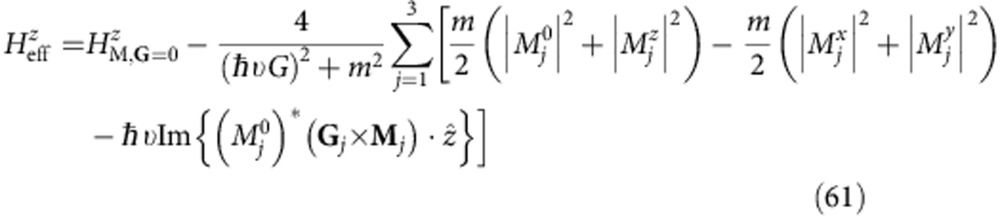


Since *m* is about an order of magnitude smaller than *ℏ*ε*G*, the second-order terms reduce approximately to those in equations (54, 55, 56, 57) and the general results of the previous section remain approximately true. Another complication is that the next shell of reciprocal lattice vectors become relevant. In this case, the symmetry properties that cause 
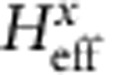
 and 
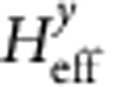
 to vanish do not strictly hold. We do, however, find that these terms are small. The primary contribution to the gap comes from the 
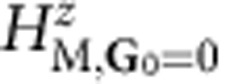
 term, which overshoots the gap. Including the second-order terms produces an excellent approximation to the calculated gap. Thus, we see explicitly that the relaxation is a key source of the gap opening in G/BN bilayer systems.

## Author contributions

J.J. and A.M.D. executed research. All authors contributed in conceiving, discussing and preparing the manuscript.

## Additional information

**How to cite this article:** Jung, J. *et al*. Origin of band gaps in graphene on hexagonal boron nitride. *Nat. Commun.* 6:6308 doi: 10.1038/ncomms7308 (2015).

## Supplementary Material

Supplementary Information Supplementary Figures 1-5, Note 1-2 and Supplementary References

## Figures and Tables

**Figure 5:**
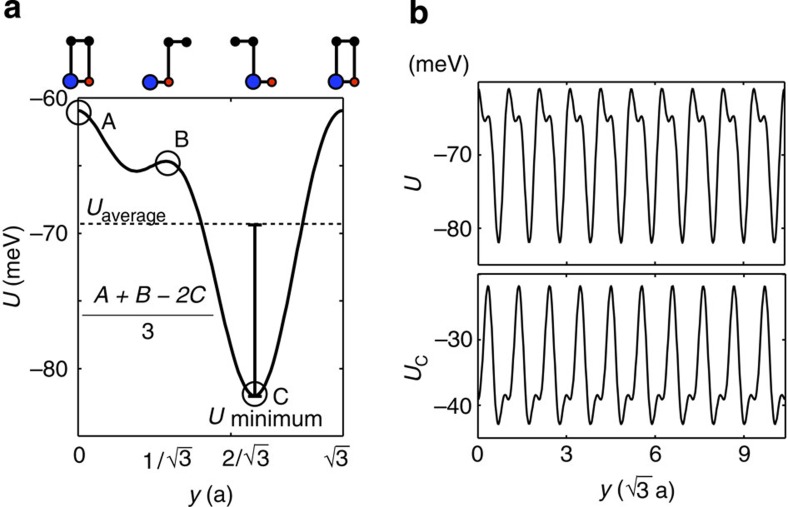
First harmonic parametrization of the interlayer interaction potential energy. (**a**) The total energy per unit cell area as a function of sliding in the *y* axis for *x*=0 shows a minimum when one of the carbon atoms sits in the middle of the hexagon and another sits on top of boron. (**b**) Potential energy of G’s carbon atoms per unit cell area. *Right bottom panel:* potential energy experienced by the individual carbon atom per unit cell area obtained assuming additivity of the energies.
