# Supplementary material for: Origin of band gaps in graphene on hexagonal boron nitride
Source: Nat Commun. 2015 Feb 19;6:6308. doi: 10.1038/ncomms7308 (PMC4346636; doi:10.1038/ncomms7308)
Supplement: Supplementary Information — Supplementary Figures 1-5, Note 1-2 and Supplementary References [file ncomms7308-s1.pdf]

Supplementary figures

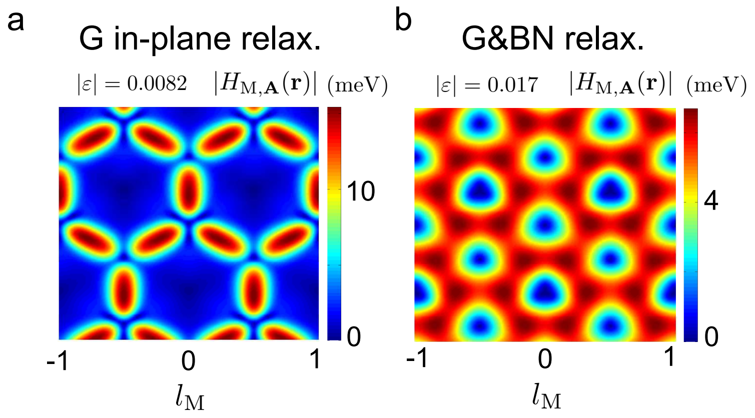

Supplementary Figure 1: Real space map of $|H_{\mathrm{M},\mathbf{A}}(\mathbf{r})|$, the pseudomagnetic field vector potential contribution to the Hamiltonian due to moiré strains for relaxed solutions. **a.** Graphene relaxation only allowing only in-plane strains with a long moiré period corresponding to $|\varepsilon| = 0.0082$. **b.** Magnitude of the vector potential contribution to the Hamiltonian due to the relaxation of the graphene sheet when both graphene and BN sheets are simultaneously allowed to relax near zero twist angle moiré period. The out of plane relaxation introduces qualitative changes in the features.

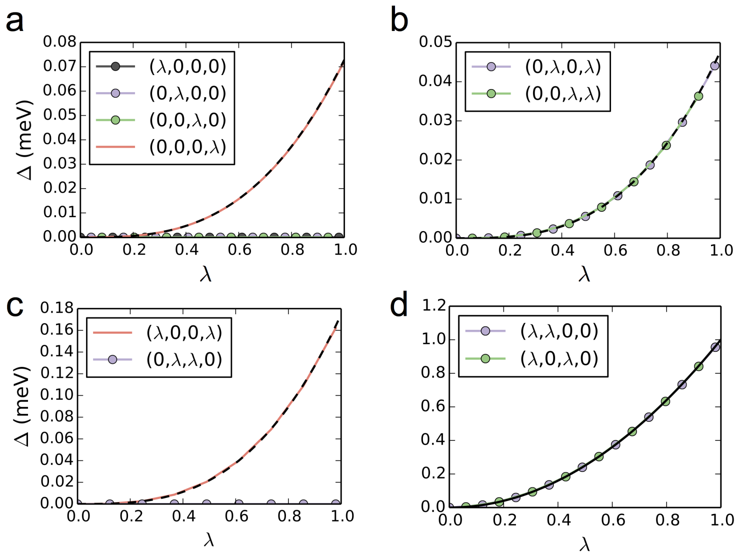

Supplementary Figure 2: **a.** Gap vs λ for individual contributions to $H_{\mathrm{M},\mathbf{G}}$ from M^0 (black dots), M^x (blue dots), M^y (green dots) and M^z (red solid line). The labels in the legend correspond to $(\lambda_0, \lambda_x, \lambda_y, \lambda_z)$. The dashed line is a fit to λ^3. **b.** Gap vs λ for contributions with both M^z and either M^x (blue) or M^y (green). The two terms are equal. Such terms contribute to H^0_{eff} at second order (see main text), but H^0_{eff} does not contribute to a gap opening. Dashed line (black) is a fit to λ^3, showing that indeed, no second order contribution is evident. **c.** Gap vs λ for contributions with both M^0 and M^z (solid red line). Such terms contribute to H^x_{eff} and H^y_{eff} at second order (see main text), which is zero due to the symmetry of the Hamiltonian. Dashed line (black) is a fit to λ^3, showing that indeed, no second order contribution is evident. Also shown (blue circles) is the contribution with both M^x and M^y, which is zero. **d.** Gap vs λ for contributions with both M^0 and either M^x (blue circles) or M^y (green circles). The two contributions are equal. Such terms contribute to h^z in the second order perturbation theory (see main text), and therefore contribute to the gap. Solid black line is a fit to λ^2, confirming the perturbation theory result.

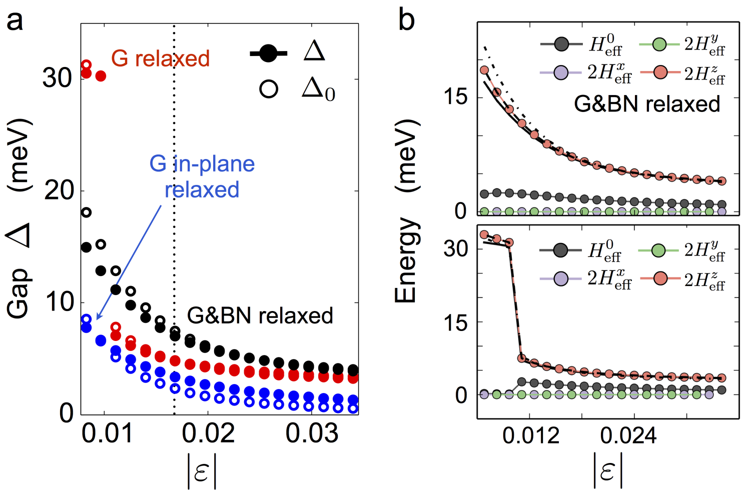

Supplementary Figure 3: Breakdown of different contributions to the single-particle band gap. **a.** Comparison of the band gap Δ represented with connected filled circles and the non-zero average contribution Δ_0 represented with empty circles for graphene relaxed, graphene and boron nitride relaxed, and restricted in-plane only relaxation of graphene. We notice that the presence of out-of-plane relaxation prevents the complete cancellation of the average mass in the presence of small in-plane strains. **b.** In the top we show the perturbation theory analysis of the gap in the configuration where graphene relaxes due to in-plane strains. The non-perturbative gap is shown in a black solid line, which is closely approximated by the 2nd order perturbation theory (dashed black line). The dash-dotted line is the gap due to the average mass. The decomposition of the Hamiltonian into $H^0_{\rm eff}$ (grey circles) and $H^\alpha_{\rm eff}$ for $\alpha = x, y, z$ (blue, green, and red circles, respectively) indicates that the primary source of the gap is $H^z_{\rm eff}$. In the bottom we show the perturbation theory analysis of the gap in the configuration where graphene and boron nitride both relax due to in-plane strains. The non-perturbative gap is shown in a black solid line, which is closely approximated by the 2nd order perturbation theory (dashed black line). The dash-dotted line is the $\mathbf{G}_0 = 0$ contribution to the gap. The decomposition of the Hamiltonian into $H^0_{\rm eff}$ (grey circles) and $H^\alpha_{\rm eff}$ for $\alpha = x, y, z$ (blue, green, and red circles, respectively) indicates that the primary source of the gap is $H^z_{\rm eff}$.

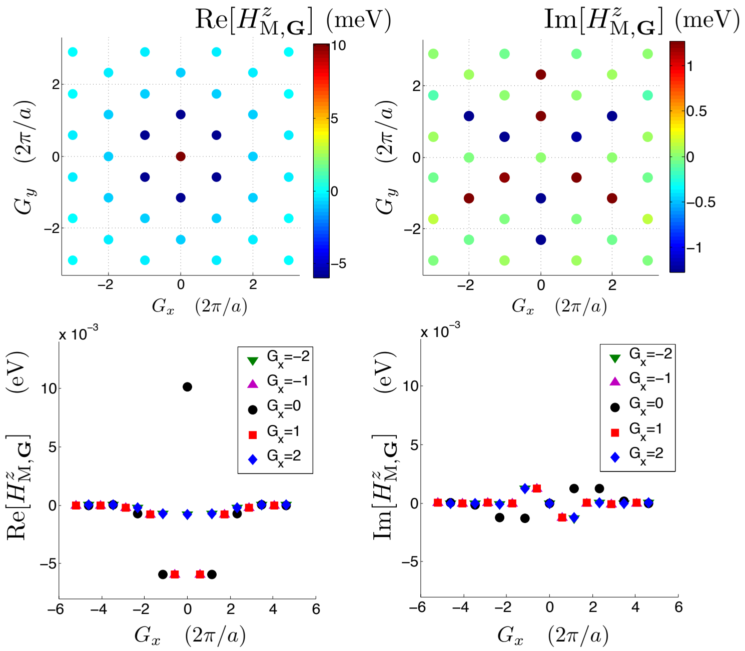

Supplementary Figure 4: A representation of the real and imaginary parts of the Fourier components of the H^z_{M} local mass term distribution in real space of the Hamiltonian for substantially strained configurations for in-plane only relaxation of the graphene sheet. The band gap is determined mainly by the average mass term from the $\mathbf{G} = (0,0)$ contribution and modified by the first hexagonal shell in $\mathbf{G}$ vectors contributing to second order in perturbation theory.

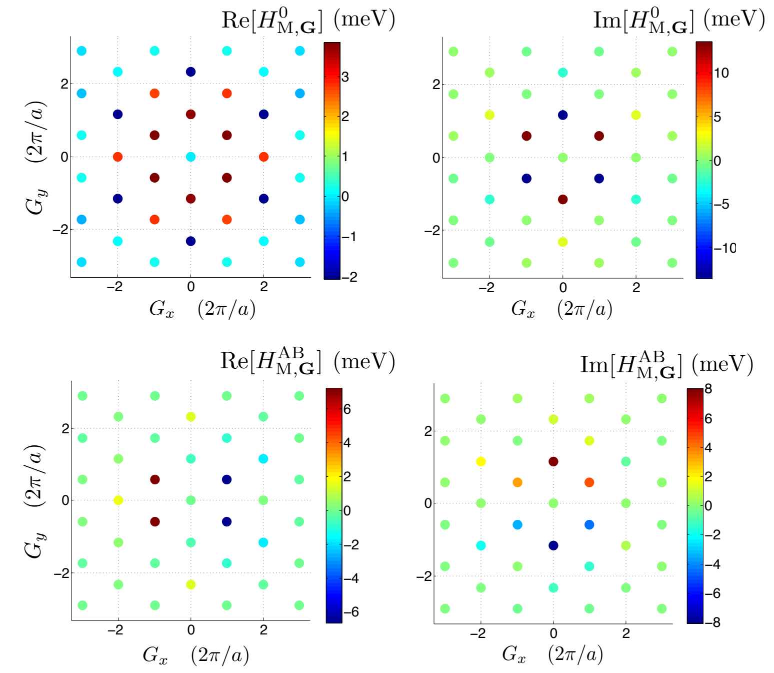

Supplementary Figure 5: Fourier expansion of the Hamiltonian for the site potential fluctuations H^0 and the anisotropic strain H^{AB} terms. The contributions to the band gap of these two terms are much smaller than those from H^z.

Supplementary Note 1: Strains in Relaxed Ground-States.

As noted in the main text the strained geometries can be characterized by the magnitude and phases that define the scalar fields and vector fields within a restricted variational space that preserves the triangular moiré periodicity dictated by the lattice constant mismatch ε and twist angle θ. The solutions for the strains in the graphene layer can be largely characterized by two parameters $C_{u,1}$ and $\varphi_{u,1}$ whereas the height profiles require three $C_{h,0}$, $C_{h,1}$ and $\varphi_{h,1}$ for the additional average interlayer separation. For the topmost BN sheet we only consider in-plane strains assuming that its separation from the additional BN layer underneath takes a constant average value. Because the relative magnitudes of the elastic and potential energies scale with the lattice constant mismatch ε a correction in the potential profiles or the average value of the elasticity constants would have an overall effect of shifting the solutions in the abscissa. Within our approximation, the solutions are completely characterized by the ε-dependent values of the parameters that define the scalar field.

When we consider the coupled graphene and BN layer relaxation we notice an interesting behavior where the largest strain magnitudes are for the topmost BN sheet rather than graphene itself. This is possible thanks to a special total energy landscape with easy sliding path in BN [1]. The BN sheets in the crystal substrate follows an AA′ stacking order and for this stacking configuration they have a minimum energy sliding path when going from AA′ to AB′ with a small barrier of about $\sim$3 meV and even smaller total energy differences of about $\sim$1 meV within the LDA. A more elaborate GGA + vdW functional calculation [1] predicted similar barrier magnitudes but with the minimum of energy happening for the AB′ stacking configuration with a total energy lower by

$\sim$1.5 meV. These minute differences are unimportant for the solutions we discuss.

Supplementary Note 2: Band Gaps and the Fourier Components of the Strained Hamiltonian.

The Hamiltonian of graphene is modified in a G/BN heterojunction by moiré patterns [2] that can be described in a transparent manner when represented in a pseudospin basis. The different contributions consist of a site potential $H^0_{\rm M}$, the mass or sublattice staggering potential $H^z_{\rm M}$ and an in-plane pseudospin inter-sublattice coherence term $H^{xy}_{\rm M} = H^{\rm AB}_{\rm M}$. The latter is closely related with a pseudomagnetic field derived from the straining of the graphene sheet represented in Supplementary Figure 3.

As mentioned in the main text and explained in more detail in the Supplementary Reference 2 it is possible to obtain the full band structure from the Fourier components represented in the moiré reciprocal lattice vectors $\mathbf{G}$. The term that most directly influences the band gap comes from $H^z_{\rm M}$, in particular the $\mathbf{G}_0 = (0,0)$ contribution which is the average mass in the a moiré supercell. This term normally vanishes to zero in a rigid crystal [2] but here we showed that they generally average to a nonzero value in the presence of in-plane strains. Farther shell contributions in $\mathbf{G}$ do also contribute to the band gap through higher order corrections. The contributions from the first shell in $\mathbf{G}$-vectors through second order perturbation theory play the most relevant role. In Supplementary Figure 4 we show a comparison of the nonzero average mass term and the total band gap as a function of moiré period. It is noteworthy that when out of plane relaxations are

absent the values of the band gaps are generally smaller and the relative cancellation between the average mass Δ_0 and the second order contributions from the first shell are more substantial. The off-diagonal term $H_{\rm M}^{\rm AB}$, due to the anisotropic strains generated by the coupling with the BN substrate, plays a minor role in configuring the band gap at the primary Dirac point. as noted in the perturbative analysis.

The other two components of the Hamiltonian, namely the potential fluctuations $H_{\rm M}^{0}$ and in-plane pseudospin terms $H_{\rm M}^{xy}$ represented in Supplementary Figure 5, also see modifications due to strains, typically acquiring contributions beyond the first shell in G-vectors with the most important contributions ranging up to three nearest neighbor hoppings, and their Fourier components having magnitudes on the order of $\sim$10 meV.

The pseudomagnetic term generated by the strains in the graphene sheet itself can in principle have a contribution comparable to the contribution due to the electron virtual hopping to and back from the BN sheet. Using the expressions for pseudomagnetic field vector potentials in the Hamiltonian of graphene provided in the Supplementary References $3, 4$

$$H^{x}_{{\rm M},\mathbf{A}}(\mathbf{r}) = g[u_{11}(\mathbf{r}) - u_{22}(\mathbf{r})] \quad (1)$$

$$H^{y}_{{\rm M},\mathbf{A}}(\mathbf{r}) = -2g u_{12}(\mathbf{r}) \quad (2)$$

and using $g \sim 1.5/a$ a typical map of its magnitude in real space is shown in Supplementary Figure 1. We note that the pseudomagnetic field vector potentials follow a moiré period scaling relation given by $|\mathbf{A}(\mathbf{r})| \propto (a/l_M) \left|\widetilde{A}(\mathbf{d}(\mathbf{r}))\right|$ when represented in rescaled coordinates of the moiré superlattice $\widetilde{A}(\mathbf{d}(\mathbf{r}))$, in turn defined by the parameters that determine the displacement vectors

$\mathbf{u}(\mathbf{r})$.

Supplementary References

1. Marom, N. *et al.* Stacking and registry effects in layered materials: The case of hexagonal boron nitride. *Physical Review Letters* **105**, 046801 (2010).

2. Jung, J., Raoux, A., Qiao, Z. & MacDonald, A. H. Ab-initio theory of moire superlattice bands in layered two-dimensional materials. *Physical Review B* **89**, 205414 (2014).

3. Suzuura H. & Ando T. & Phonons and electron-phonon scattering in carbon nanotubes. *Physical Review B* **65**, 235412 (2002).

4. Vozmediano, M., Katsnelson, M. & Guinea, F. Gauge fields in graphene. *Physics Reports* **496**, 109–148 (2010).
